# Perinatal depressive and anxiety symptoms are associated with gut microbiota in pregnant women with overweight and obesity

**DOI:** 10.1016/j.bbih.2025.101042

**Published:** 2025-06-19

**Authors:** Janina Hieta, Chouaib Benchraka, Katariina Pärnänen, Noora Houttu, Kati Mokkala, Mrunalini Lotankar, Eeva-Leena Kataja, Leo Lahti, Kirsi Laitinen

**Affiliations:** a Integrative Physiology and Pharmacology Unit, Institute of Biomedicine, Faculty of Medicine, University of Turku, Finland; b Nutrition and Food Research Center, Faculty of Medicine, University of Turku, Turku, Finland; c Department of Computing, Faculty of Technology, University of Turku, Turku, Finland; d The FinnBrain Birth Cohort Study, Turku Brain and Mind Center, Department of Clinical Medicine, University of Turku, 20520, Turku, Finland; e Centre for Population Health Research, University of Turku and Turku University Hospital, 20520, Turku, Finland; f Department of Obstetrics and Gynecology, Turku University Hospital, Wellbeing Services County of Southwest Finland, Turku, Finland

**Keywords:** Gut microbiota, Pregnancy, Overweight, Depression, Anxiety, Gut-brain axis

## Abstract

The associations of gut microbiota with depressive and anxiety symptoms have been investigated mainly in non-pregnant humans, and currently there is a significant gap in research on pregnant women, especially those who are living with overweight and thus at a higher risk for experiencing perinatal mental health problems. In this study, we used shotgun metagenomic sequencing to analyze the gut microbiota of pregnant women with overweight and obesity, both in early and late pregnancy. We compared gut microbial diversity, composition, and function across groups with different trajectories of depressive (n=419) and anxiety (n=408) symptoms. Depressive symptoms were assessed using the Edinburgh Postnatal Depression Scale (EPDS), and anxiety symptoms were evaluated with the Symptom Checklist 90 (SCL-90, anxiety subscale) at five time points spanning from early pregnancy to one year postpartum. Latent growth mixture modeling (LGMM) was used to model symptom trajectories from early pregnancy until one year postpartum and further symptom sum scores at five time points cross-sectionally. We observed differences in several bacterial species abundances between the trajectory groups and in cross-sectional analyses, including higher abundance of *Hungatella hathewayi* in the Moderate and increasing depressive symptoms group (FDR<0.25), and *Bacteroides clarus* in the High and decreasing depressive symptoms group (FDR<0.25) and in women experiencing clinically significant postpartum anxiety symptoms (FDR<0.05). No differences were found regarding the gut microbiota diversity (α or β) or function. The results suggest that maternal gut microbiota, particularly the increased abundance of possible pro-inflammatory species, could be one of the factors affecting perinatal distress.

## Highlights

• Women with perinatal depressive and anxiety symptoms showed differences in the gut microbiota composition.
• Higher abundances of *Hungatella hathewayi* were found in women with postpartum depressive symptoms.
• Higher levels of *Bacteroides clarus* were found in women with prenatal depressive and postpartum anxiety symptoms.

## 1 Introduction

Perinatal depression and anxiety refer to mood conditions that occur during pregnancy and/or within the first 12 months of the postpartum period. These symptoms may have several adverse impacts on pregnancy outcomes and on the newborn’s health. For example, prenatal depression has previously been linked to preterm birth (Miller et al., 2022), and a reduced birth weight (Li et al., 2020). Maternal symptoms can also adversely impact detrimentally on early infant neurodevelopment, potentially impacting on communication abilities and gross motor skills (Zhang et al., 2023). Pregnant women with overweight or obesity is a study population of particular interest since they are a risk group for various obstetric complications (Dreisbach et al., 2019). It has also been reported that women with overweight or obesity have an increased risk for developing depression in the postpartum period (Ertel et al., 2017). Thus, there is a need for gaining a deeper understanding of the mechanisms of perinatal mental health conditions, especially in this high-risk group. The previous research, which have included only pregnant women with normal weight suggest that one possible mechanism may be traced to the gut-brain axis (GBA) and the ability of gut microbiota to influence cerebral functions through various pathways (Xie et al., 2024).

Recent research has revealed that the gut microbiota is associated with depression and other mental health conditions, highlighting the importance of the GBA (Simpson et al., 2021). While the diversity (α and β diversity) and composition of gut microbiota have been demonstrated to differ between individuals with depression or anxiety and healthy controls in non-pregnant populations, (Jiang et al., 2018; Kelly et al., 2016; Mason et al., 2020; Vodička et al., 2018), there are only a limited number of studies which have examined differences in gut microbial populations associated with perinatal depression and anxiety. In one study, no significant differences in α- or β-diversity were observed with respect to depressive symptoms, but a higher abundance of *Candidatus Soleaferrea* was associated with a lower risk of depressive symptoms during pregnancy (Xie et al., 2024). Similarly, notable differences in the microbiota composition during pregnancy were detected between women with postpartum depression and those not suffering from these symptoms. In that study, depressive symptoms were associated specifically with higher levels of *Shigella*, an Enterobacteriaceae member (Zhou et al., 2020). This bacterium is known to be able to induce gut inflammation and increase gut wall permeability, which may in turn evoke a systemic inflammation (Zhou et al., 2020). In addition, our previous study demonstrated that a probiotic and/or fish oil intervention had a modest effect on depressive symptoms in pregnant women (Hulkkonen et al., 2021), with one possible mechanism being that these symptoms were mediated by the gut microbiota.

It has been reported that depressive and anxiety symptoms fluctuate during pregnancy and in the postpartum period (Korja et al., 2018). Thus, we primarily focused on examining the GBA by investigating gut microbiota associations with trajectories of depressive and anxiety symptoms. The aim was to determine whether there would be differences in the gut microbiota composition, diversity, and function between subjects in the different symptom trajectories (Hulkkonen et al., 2021). The subjects were examined in the perinatal period of early pregnancy up to 12 months postpartum. Further, we aimed to investigate the relationship between the gut microbiota during pregnancy and the subjects’ depressive and anxiety symptoms at five individual measurement points during and after pregnancy. We applied shotgun metagenomic sequencing in preference over 16S rRNA sequencing as it provides more detailed information about the gut microbiota at the species level and the functional capabilities of the gut microbiota.

## 2 Methods

### 2.1 Study design

We investigated the relationship of gut microbiota to perinatal depressive and anxiety symptoms in pregnant women with overweight and obesity enrolled in a mother-child dietary intervention trial (ClinicalTrials.gov NCT01922791) conducted in Southwest Finland (n=439). The recruitment for the trial was between October 2013–July 2017. The Ethics Committee of the Hospital District of Southwest Finland approved the study protocol, and all participants provided written informed consent. The original study protocol has been previously reported in detail (Pellonperä et al., 2019). Briefly, the original intervention study aimed to investigate in a double-blind, placebo-controlled setting, whether the fish oil and/or probiotics could decrease the risk of gestational diabetes mellitus. Inclusion criteria were overweight (body mass index, BMI ≥25 kg/m^2^), early pregnancy (<18gestational weeks), and the absence of chronic diseases. Exclusion criteria included individuals with preexisting diabetes (HbA1c ≥ 6.5 % [48 mmol/mol] or fasting glucose ≥7.0 mmol/L at randomization), those with multifetal pregnancies, and subjects with chronic conditions that could influence metabolic and gastrointestinal health. As the study was not powered for the predefined outcome variables of this report, the intervention is used as a covariate in this exploratory longitudinal study.

**Fig. 1:**
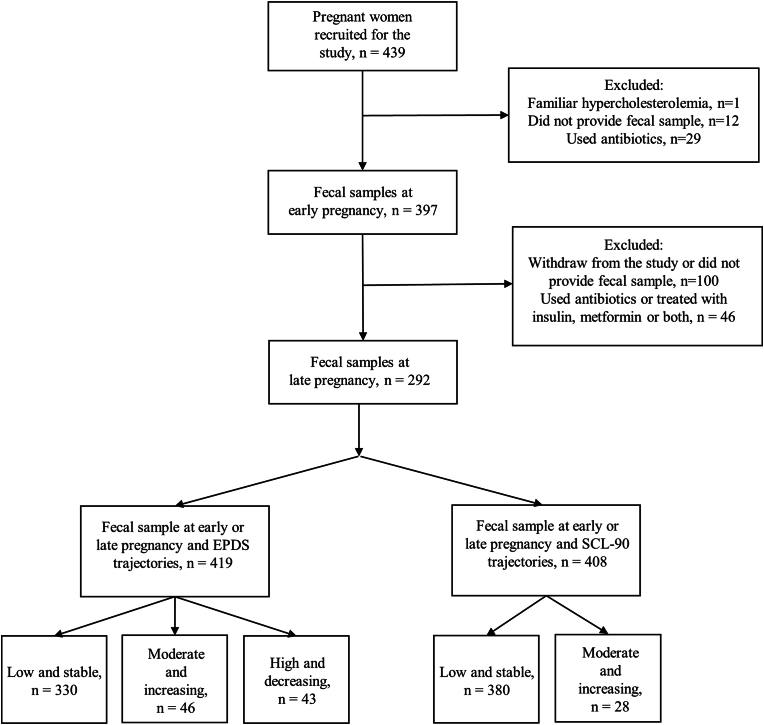
The flowchart of the present study.

In the present study, the participants who had used antibiotics within eight weeks before fecal sample collection and women who did not provide a fecal sample during early (mean: 13.8 +/- 2.1 gestational weeks) or late (mean: 35.2 +/- 0.9 gestational weeks) pregnancy or were treated with metformin or insulin during late pregnancy were excluded (Fig. 1).

### 2.2 Depressive and anxiety symptoms and clinical parameters

Participants were assessed for depression and anxiety symptoms during early and late pregnancy, as well as at three, six, and 12 months postpartum. Depressive symptoms were measured using the 10-item Edinburgh Postnatal Depression Scale (EPDS; Cox et al., 1987), with a score range of 0–30. Maternal prenatal and postnatal anxiety symptoms were assessed using the 10-item Symptom Checklist 90 (SCL-90; Derogatis and Unger, 2010), with scores ranging from 0 to 40. Latent Growth Mixture Modeling (LGMM) was used in Mplus (Muthén, 1998) to identify latent trajectory groups for each symptom category. Individual item scores were used in the models, and participants with missing data were included using maximum likelihood under the missing-at-random assumption. The optimal number of latent trajectory groups was determined by comparing model fit indices (BIC, posterior probability, and entropy). For depression, a three-group solution was used: "Low and stable", "Moderate and increasing," and "High and decreasing." For anxiety, two groups were identified: "Low and stable" and "Moderate and increasing". A more detailed description of the method and the selected model has been presented previously (Hulkkonen et al., 2021). In addition to trajectory analyses, we also conducted cross-sectional analyses using individual EPDS and SCL-90 sum scores in early and late pregnancy, as well as at three, six, and 12 months postpartum. For the categorical EPDS and SCL-90 variables a cut-off value of >10 was used to indicate clinically significant depressive and anxiety symptoms. The cut-off values were determined based on the previous studies conducted in the same geographical area (Mathiasen et al., 2023; Sirkiä et al., 2024). The percentage of women scoring above the cut-off for depressive symptoms ranged from 9.7 % to 12.4 % at different time points (early and late pregnancy, and at 3, 6, and 12 months postpartum) with the corresponding percentage for anxiety symptoms ranging from 3.1 % to 5.4 % (Supplementary Table S1).

The height of participants was measured with a wall stadiometer and self-reported weight was obtained from maternal welfare clinic records. Pre-pregnancy BMI was determined using height and self-reported pre-pregnancy weight from clinical records. A BMI ≥25 < 30 kg/m^2^ was considered overweight, while a BMI ≥30 kg/m^2^ was classified as obesity. Daily dietary intake was calculated from 3-day food diaries with appropriate software (AivoDiet 2.0.2.3; Aivo, Turku, Finland). The participants received written instructions and a template to record all foods and beverages consumed over three days. The index of dietary quality (IDQ) which assess the overall dietary quality of the diet was calculated as previously described (Leppälä et al., 2010). The information on education was collected from a questionnaire and during an interview.

### 2.3 Fecal samples and metagenomic sequencing

Fecal samples were gathered in clean, sterile plastic containers either in the morning of the study visit or on the night before, during both early and late pregnancy. The samples were then stored at a temperature of −20 °C until DNA extraction. The metagenomics sequencing was done with the Illumina HiSeq-platform using paired-end sequencing in Clinical Microbiomics (Denmark). DNA extraction, sequencing, quality control and removing human sequences have been described in detail previously (Mokkala et al., 2021). A taxonomic classification was performed using MetaPlAlan v4.0.1 (Blanco-Míguez et al., 2023) with a database spanning 26,970 species-level genome bins (Pasolli et al., 2019); using the default settings. Functional profiles were obtained using HUMAnN v3.0.1 (Beghini et al., 2021) using the full pangenome and protein (UniRef90) database provided; ran with the default settings as well. Both analyses were performed using a distributed cloud computing environment (CSC) with the help of snakemake v7.6.1 (Mölder et al., 2021).

### 2.4 Statistical analyses

The statistical software R version 4.2.1 was used to perform the analyses. The relationship between the maternal gut microbiota and depressive and/or anxiety symptoms during pregnancy up to 12 months postpartum was analyzed using measures of α- and β-diversity and differential abundances. The α- and β-diversities were investigated at the species level. α-Diversity was investigated by using the Shannon index and the difference between study groups was statistically evaluated with linear model adjusted with covariates; prepregnancy BMI, intervention group (except for analysis with early pregnancy gut microbiota and depressive/anxiety symptoms), Index of Diet Quality (IDQ) and smoking status before pregnancy. Covariates were selected based on the group differences between depressive and anxiety symptom trajectories (presented in Table 1) or existing knowledge about factors (prepregnancy BMI, IDQ) that influence depressive and anxiety symptoms or gut microbiota composition (Dreisbach et al., 2019; Lotankar et al., 2022). β-Diversity was analyzed using the Bray-Curtis dissimilarity index and visualization was done with Principal Coordinates Analysis (PCoA/MDS) using the mia v1.8.0 (Ernst et al., 2023) R package. The permutational analysis of variance test (PERMANOVA) from the R vegan package (Oksanen et al., 2024) v2.6-4 adjusted with the covariates was used to test the statistical significance of the results with a p-value<0.05 considered statistically significant. Differential abundances at the species levels, as well as the functional data, were examined with MaAsLin2 v1.15.1 (Mallick et al., 2021) with a prevalence of 10 % and detection limit on relative abundance data of 1e-4. FDR-corrected p-value<0.05 was considered statistically significant and FDR-corrected p-value<0.25 borderline statistically significant.

**Table 1:** Clinical characteristics of the participating women according to the anxiety and depressive symptom trajectories.

Clinical characteristics	All	n	Anxiety symptom trajectories		p-value	n	Depressive symptom trajectories			p-value
	n = 419		Low and stable	High and decreasing			Low and stable	Moderate and increasing	High and decreasing	
Age (y)^1^	30.6±4.5	380/28	30.7±4.6	31.6±4.2	0.3	330/46/43	30.8±4.5	29.4±3.9	30.8±5.2	0.1
Prepregnancy BMI (kg/m2)^2^	28.7 (26.5–32)	380/28	29.0 (26.5–32.2)	27.77 (26.2–29.5)	0.1	330/46/43	28.7 (26.5–31.9)	28.2 (26.3–30.7)	29.4 (26.3–33.4)	0.3
Overweight	254 (60.6)		225 (59.2)	22 (78.6)	0.05		197 (59.7)	33 (71.7)	24 (55.8)	0.2
Obese	165 (39.4)		115 (40.8)	6 (39.5)			133 (40.3)	13 (28.3)	19 (44.2)	
University degree^3^	233 (55.6)	345/26	218 (63.2)	12 (46.2)	0.08	303/46/30	195 (64.4)	23 (50)	15 (50)	0.07
Smoking before pregnancy^2^	81 (19.3)	347/26	71 (20.5)	8 (30.8)	0.2	305/46/30	57 (18.7)	13 (28.3)	11 (36.7)	**0.03**
Smoking during pregnancy^2^	18 (4.3)	345/26	15 (4.3)	3 (11.5)	0.1	303/46/30	11 (3.6)	4 (8.7)	3 (10)	0.1
MET index		377/27			0.8	328/45/42				0.3
Light	213 (51.3)		192 (50.9)	14 (51.9)			159 (48.5)	29 (64.4)	25 (59.5)	
Moderate	174 (41.9)		159 (42.2)	12 (44.4)			145 (44.2)	14 (31.1)	15 (35.7)	
Vigorous	28 (6.7)		26 (6.9)	1 (3.7)			24 (7.3)	2 (4.4)	2 (4.8)	
IDQ^2^		378/28			0.6	328/46/43				0.3
Poor	219 (52.3)		196 (51.9)	16 (57.1)			166 (50.6)	27 (58.7)	26 (60.5)	
Good	198 (47.3)		182 (48.1)	12 (42.9)			162 (49.4)	19 (41.3)	17 (39.5)	
Dietary intake										
Energy (kJ)	8133.5±1951.5^1^	370/28	8172.6±1952.2^1^	7488±2029.2^1^	0.2	321/44/41	8095 (6979.5–9274.25)^2^	7760 (6454–9899)^2^	7310 (6012.75–9011.5)^2^	0.2
Fat (g)	78.2±25.5^1^	370/28	78.3±25.6^1^	75.65±26.4^1^	0.6	321/44/41	77.5 (62.2–93.3)^2^	80.7 (59.9–94.7)^2^	71.8 (57.9–89.6)^2^	0.5
Fiber (g)	17.8±6.2^1^	370/28	17.9±6.1^1^	16.65±7.2^1^	0.3	321/44/41	17.2 (13.7–21.3)^2^	17.4 (13.1–24)^2^	14.4 (13–19.8)^2^	0.07

BMI = Body Mass Index, IDQ = Index of Diet Quality.

^1^mean ± SD (standard deviation), ^2^median (IQR, interquartile range) or ^3^frequency (%).

Independent sample *t*-test or One-way ANOVA for normally distributed variables, Mann-Whitney or Independent Kruskal-Wallis test for non-normally distributed variables, and the chi-squared test for categorical variables. p < 0.05 is considered statistically significant.

Data describing the clinical characteristics of the participants were analyzed using SPSS Statistics 29.0 (IBM, Chicago, IL, USA). The skewness value was used to determine the normality (<1) of the data. Normally distributed continuous variables are presented as the mean and standard deviation, while those not normally distributed are reported as the median and interquartile range. Categorical variables are described in terms of frequency and percentage. In the comparisons, one-way ANOVA and independent-samples *t*-test were applied if the data were normally distributed, while the Kruskal-Wallis test or Mann–Whitney *U* test was used for non-normally distributed data. The relationships between categorical variables were explored using cross-tabulation, and the chi-squared test was performed to test the significance of these relationships. A p-value<0.05 was considered statistically significant.

## 3 Results

### 3.1 Clinical characteristics

The clinical characteristics of the participants at baseline are presented in Table 1. Of the participating women, 60.6 % were identified as with overweight, while 39.4 % were classified as with obesity. The majority of the women (55.6 %) had a university degree. The EPDS trajectories differed based on the prepregnancy smoking status (p=0.03). Specifically, the frequency of smoking before pregnancy was highest in the High and decreasing group and lowest in the Low and stable group. The postpartum clinical characteristics of the participants according to depressive and anxiety symptom trajectories are presented in the Supplementary Table S2.

### 3.2 The association between species abundances and perinatal depressive and anxiety symptom trajectories

**Fig. 2:**
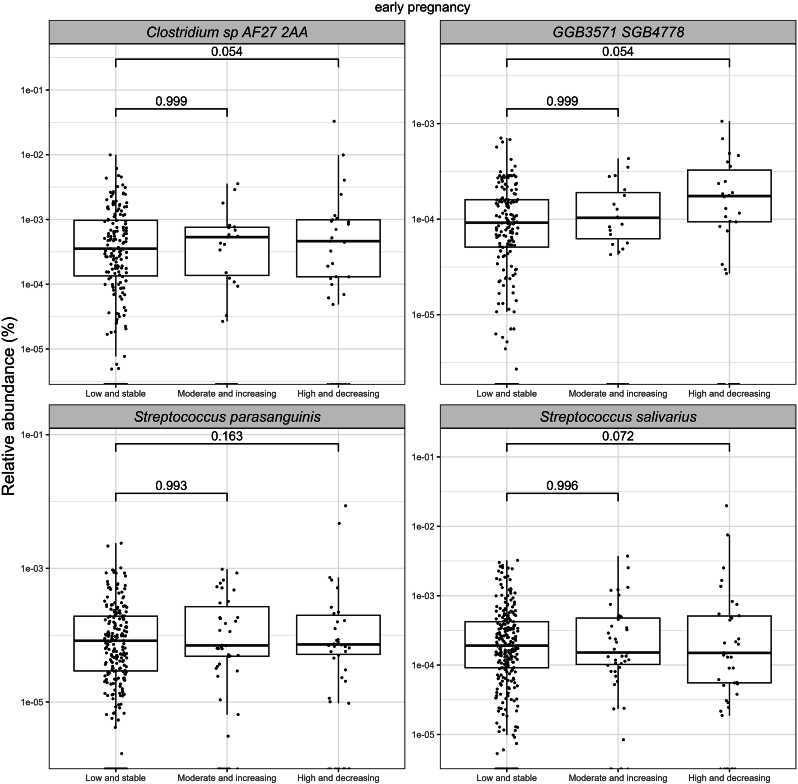
A comparison of relative abundances of bacterial species with borderline significant (FDR<0.25) differences in early pregnancy between women with different depressive symptom trajectories from early pregnancy up to 12 months postpartum. Each dot in the boxplot represents a single observation. The significance was estimated with MaAsLin2 with a prevalence of 10 % and detection limit on relative abundance data of 1e-4. The following covariates were included in the model: prepregnancy BMI, intervention, index of diet quality and smoking status before pregnancy.

In early pregnancy, higher abundances of four bacterial species, namely *Clostridium* sp*.* AF27-2AA, GGB3571 SGB4778*, Streptococcus parasanguinis, and S. salivarius* (FDR<0.25), were observed in the EPDS trajectories of the High and decreasing group as compared to the Low and stable group (Fig. 2; Supplementary Table S3). In terms of the SCL-90 trajectories, no differences in relative bacterial abundances between the groups were detected in early pregnancy.

**Fig. 3:**
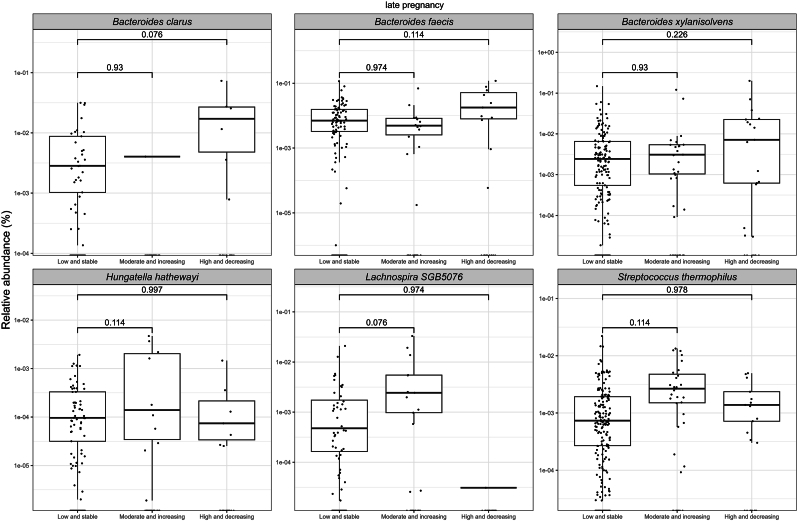
A comparison of relative abundances of bacterial species with borderline significant (FDR<0.25) differences in late pregnancy between women with different depressive symptom trajectories from early pregnancy up to 12 months postpartum. Each dot in the boxplot represents a single observation. The significance was estimated with MaAsLin2 with a prevalence of 10 % and detection limit on relative abundance data of 1e-4. The following covariates were included in the model: prepregnancy BMI, intervention, index of diet quality and smoking status before pregnancy.

**Fig. 4:**
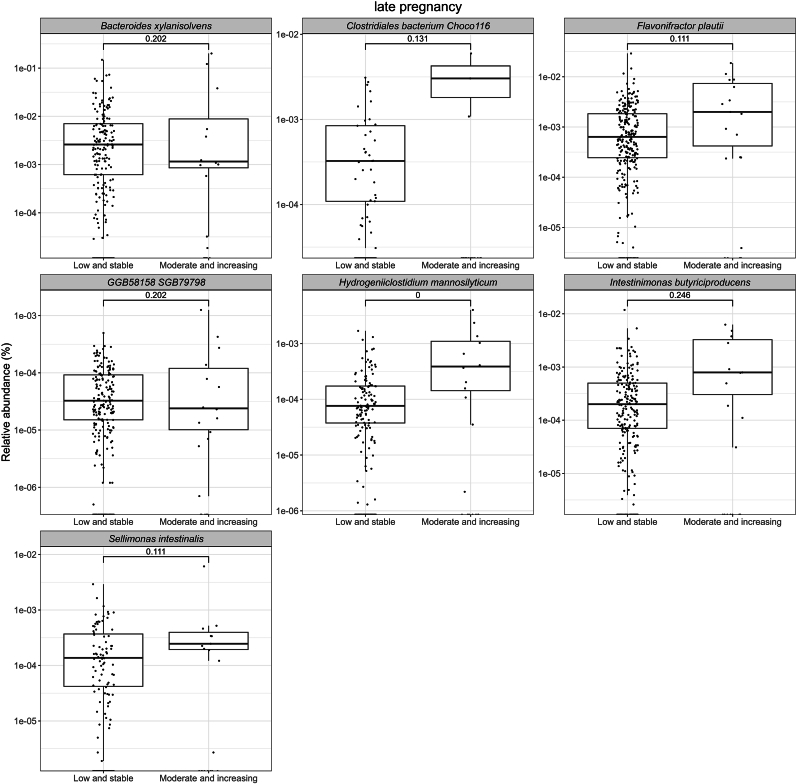
A comparison of relative abundances of bacterial species with significant (FDR<0.05) or borderline significant (FDR<0.25) differences in late pregnancy between women with different anxiety symptom trajectories from early pregnancy up to 12 months postpartum. Each dot in the boxplot represents a single observation. The significance was estimated with MaAsLin2 with a prevalence of 10 % and detection limit on relative abundance data of 1e-4. The following covariates were included in the model: prepregnancy BMI, intervention, index of diet quality and smoking status before pregnancy.

In late pregnancy, the relative abundances of *Bacteroides clarus, B. faecis,* and *B. xylanisolvens* were higher in the EPDS High and decreasing group, and those of *Hungatella hathewayi, Lachnospira* SGB5076*,* and *Streptococcus thermophilus* were higher in the EPDS Moderate and increasing group (FDR<0.25) as compared to the Low and stable group (Fig. 3; Supplementary Table S4). With respect to the anxiety symptoms, we observed higher relative abundances of *Hydrogeniiclostidium mannosilyticum* (FDR<0.05)*, B. xylanisolvens, Clostridiales bacterium* Choco116*, Flavonifractor plautii,* GGB58158 SGB79798*, Intestinimonas butyriciproducens, and Sellimonas intestinalis* (FDR<0.25) in the SCL-90 Moderate and increasing group compared to the Low and stable group (Fig. 4; Supplementary Table S5).

When we examined the changes in bacterial abundances from early to late pregnancy within the EPDS and SCL-90 trajectory groups, we identified both statistically significant and borderline significant changes in four species in the EPDS trajectory groups. In the EPDS High and decreasing group, the shift from early to late pregnancy was associated with an increase in the abundance of the *B. clarus* (FDR<0.25) and a decrease in the abundance of *Clostridium* sp. AF27-2AA (FDR<0.05). In the EPDS Moderate and increasing group, the change from early to late pregnancy was associated with an increased abundance of *Lachnospira* SGB5076 (FDR<0.05) and *S. thermophilus* (FDR<0.25) (Supplementary Fig. S1; Supplementary Table S6).

### 3.3 Relationship between species abundances during pregnancy and prenatal depressive and anxiety symptoms

We observed several statistically significant and borderline statistically significant differences in the gut microbiota in women who had clinically significant depressive and anxiety symptoms (Categorical variable with a cutoff>10 was applied) compared to those who did not have these symptoms at any of the individual time points during pregnancy. The higher relative abundances of the species GGB9634 SGB15093 in early pregnancy gut microbiota was associated with clinically significant depressive symptoms (Categorical variable with a cutoff>10 was applied) in late pregnancy (FDR<0.05) (Supplementary Table S7). In late pregnancy gut microbiota, higher abundances of one bacterial species, GGB9623 SGB15076 were significantly (FDR<0.05) associated with depressive symptoms at that time (Supplementary Table S8).

During early pregnancy, we observed higher abundances of four species in women with clinically significant anxiety symptoms in early pregnancy and three species in women with anxiety symptoms in late pregnancy (Supplementary Table S10). However, all of the observed species differed only borderline significantly between groups. During late pregnancy, the relative abundances of bacterial species *Anaeromassilibacillus* sp*.* An250*,* GGB3523 SGB4703, GGB9633 SGB15090*, Parabacteroides goldsteinii, and Ruminococcaceae* unclassified SGB15265 were significantly higher (FDR<0.05) in women with clinically significant anxiety symptoms (Supplementary Table S11). Finally, the abundances of five bacterial species increased during the shift from early to late pregnancy among women with clinically significant anxiety symptoms in late pregnancy; of those, only species *Candidatus Borkfalkia ceftriaxoniphila* and *Clostridia unclassified* SGB4447 increased significantly (FDR<0.05). The abundances of five species also decreased during the shift from early to late pregnancy among women with clinically significant anxiety symptoms in late pregnancy, and species GGB9633 SGB15090 and *P. goldsteinii* differed significantly (FDR<0.05) (Supplementary Table S12).

### 3.4 Relationship between species abundances during pregnancy and postnatal depressive and anxiety symptoms

When focusing on postnatal depressive and anxiety symptoms (Categorical variable with a cutoff >10 was applied), we observed several statistically significant and borderline statistically significant differences in the relative abundances of gut microbial species during pregnancy. A higher abundance of eight bacterial species in late pregnancy were associated with clinically significant depressive symptoms at three months postpartum, but species GGB3277 SGB4327 and GGB58158 SGB79798 remained significant after FDR correction (FDR<0.05) (Supplementary Table S8). Higher abundance of species *H. hathewayi* was (FDR<0.05) associated with clinically significant depressive symptoms at six months postpartum (Supplementary Table S8). Additionally, the relative abundances of *I. butyriciproducens* (FDR<0.05*)* decreased, while the relative abundance of *H. hathewayi* (FDR<0.25*)* increased during pregnancy in women with clinically significant depressive symptoms at the six months postpartum assessment (Supplementary Table S9).

Higher abundances of nine species in late pregnancy were associated with clinically significant anxiety symptoms at three months postpartum, but only the relative abundances of species GGB58158 SGB79798, GGB9537 SGB14940, *H. mannosilyticum* and *R. callidus* differed (FDR<0.05) between the groups (Supplementary Table S11). Additionally, the higher abundances of total 21 species in late pregnancy were associated with clinically significant anxiety symptoms at six months. Among these, *B. clarus, Bifidobacterium pseudocatenulatum, Blautia massiliensis, Clostridium spiroforme, Eubacteriaceae bacterium, F. plautii,* GGB9494 SGB14891, GGB9627 SGB15081, *H. mannosilyticum, R. torques*, and *S. intestinalis* exhibited higher abundances in the women experiencing clinically significant anxiety symptoms at six months postpartum (FDR<0.05) (Supplementary Table S11). Higher abundances of 17 species were associated with clinically significant anxiety symptoms at 12 months postpartum. Among these, species *B. uniformis, C. spiroforme, E. bacterium, F. plautii,* GGB9347 SGB14313*, H. mannosilyticum, R. callidus,* and *S. intestinalis*, between-groups comparisons revealed a significant difference at a level below FDR<0.05.

The change in abundances of 21 species during pregnancy were significantly or borderline significantly associated with clinically significant anxiety symptoms at three, six, or 12 months postpartum (Supplementary Table S12). The species that were statistically significantly associated with associated with anxiety symptoms at three months postpartum were GGB58158 SGB79798 and GGB9537 SGB14940 (FDR<0.05). The species *C. spiroforme, Clostridium symbiosum, F. plautii*, GGB9537 SGB14940, and *H. mannosilyticum* were associated (FDR<0.05) with anxiety symptoms at six months postpartum, and those of *Clostridium* sp. AF27-2AA, *C. spiroforme*, C*. symbiosum, F. plautii,* and *H. mannosilyticum* were (FDR<0.05) associated with anxiety symptoms at 12 months postpartum.

Some of the bacteria mentioned above were also found in the cross-sectional analyses with continuous variables for depression and anxiety (Supplementary table S13 A-F); however, *Collinsella aerofaciens* (FDR<0.25) was associated with the increased EPDS scores at three months postpartum (Supplementary table S13 B).

### 3.5 Gut microbiota diversity and perinatal depression and anxiety symptoms

**Fig. 5:**
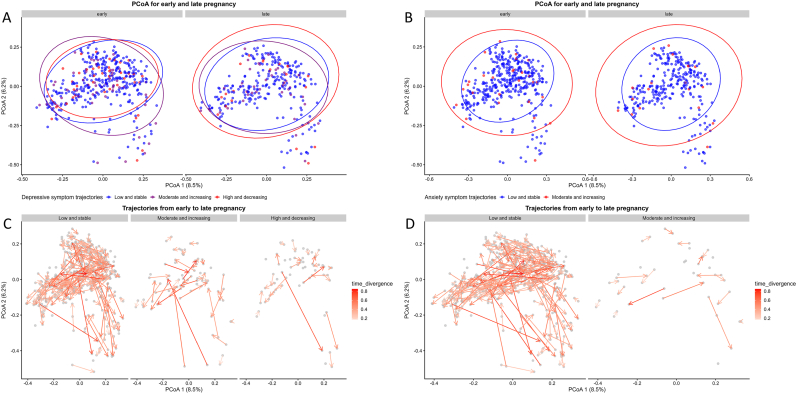
A, PCoA in early and late pregnancy at species level. Each point corresponds to a study participant and is colored to represent the following groups; blue for the women with low and stable depressive symptoms, violet for the women with moderate and increasing depressive symptoms and red for the women with high and decreasing depressive symptoms, B, blue for the women with low and stable anxiety symptoms and red for the women with moderate and increasing anxiety symptoms. C, Trajectories from early to late pregnancy within the depressive symptom trajectory groups. D, Trajectories from early to late pregnancy within the anxiety symptom trajectory groups. (For interpretation of the references to color in this figure legend, the reader is referred to the Web version of this article.)

The α-diversity (Shannon index) in early or late pregnancy, or its change from early to late pregnancy, was not associated with either the EPDS (early pregnancy p=0.4, late pregnancy p=0.3, change p=0.2; linear model) or the SCL-90 (early pregnancy p=0.2, late pregnancy p=0.4, change p=0.2; linear model) trajectories (Supplementary Figs. S2–S5). In addition, no differences were found in the Shannon index when analyzing depression and anxiety symptoms as categorical (based on cut-off value of 10) and continuous variables across each time point from early pregnancy up to 12 months postpartum (data not shown). An evaluation of the β-diversity visualized by Principal Coordinates Analysis (PCoA) utilizing Bray–Curtis dissimilarity did not reveal any distinctions between the EPDS or SCL-90 trajectory groups (Fig. 5). Moreover, we found no associations between β-diversity and depressive and anxiety symptoms when analyzed at the individual time points.

### 3.6 Gut microbial function

We observed several functional pathways in early and late pregnancy that were associated with depressive and anxiety symptom trajectories as well as symptoms evaluated at the individual time points (Supplementary table S14 A-H). The functional pathways were primarily related to bacterial housekeeping properties, for example, energy and vitamin metabolism.

## 4 Discussion

This longitudinal study revealed that the maternal gut microbiota composition, particularly the relative abundances of specific bacteria, differed between women exhibiting trajectories of either High and decreasing or Moderate and increasing depressive and anxiety symptoms, compared to those with consistently Low and stable symptom trajectories during the perinatal period. A further analysis, utilizing symptom scores and cut-off values to indicate clinically significant depressive and anxiety symptoms at individual time points, revealed differences in several bacterial abundances in the women’s gut microbiota. These findings partially aligned with the results obtained from trajectory analysis. Nonetheless, no differences were detected in α- or β-diversity in any symptoms or symptoms trajectories.

As far as we are aware, this is the first study that has investigated the relationship between gut microbiota composition and function during pregnancy and perinatal depressive and anxiety symptoms over a period that extended to one year postpartum and utilizing symptom trajectories. We observed a higher abundance of *B. clarus* in late pregnancy in women with High and decreasing depressive symptom trajectories. Additionally, the abundance of *B.clarus* was found to be higher in women with clinically significant anxiety symptoms at six months postpartum. These findings suggest that *B. clarus* is associated with both prenatal depressive symptoms, as well as postnatal anxiety symptoms. One proposed mechanism of the GBA suggests that inflammation and pro-inflammatory molecules act as mediators within the GBA (Morais et al., 2020). Thus, we speculate that one possible mechanism to explain how *B. clarus* may impact on depressive and anxiety symptoms i.e., this bacterium possesses an inflammatory potential as it was previously shown to be related to a pro-inflammatory diet (Costa et al., 2022) and a diet with an inflammatory potential has been associated with postpartum depression (Zou et al., 2022). Pro-inflammatory diet has been also linked to higher risk for total anxiety disorder in a prospective study in non-pregnant women (Zheng et al., 2024).

Another interesting finding is an enrichment of the species *H. hathewayi* in women with postpartum depressive symptoms. This is in accord with a previous study where a higher abundance of *H*. *hathewayi* was found to be associated with major depressive disorder in non-pregnant individuals (Maes et al., 2023). *H. hathewayi* has been described as a “possible pathogen” in humans, since a higher abundance has been associated with colorectal cancer (Z. Huang et al., 2023) and multiple sclerosis (X. Zhou et al., 2022). Regarding the *Hungatella* genera, an association has been observed between *Hungatella* and symptoms of depression in non-pregnant individuals (Radjabzadeh et al., 2022). The possible mechanism behind *H. Hatheway’s* properties may be related to its capacity to produce trimethylamine-N-oxide (TMAO), a compound linked with some neurological diseases including depression (Mudimela et al., 2022). Previous research shows that circulating TMAO activates pro-inflammatory signaling pathways and thus, may induce the neuroinflammation (Brunt et al., 2020). Furthermore, we observed that a higher abundance of *S. intestinalis* in late pregnancy would be associated with both postpartum anxiety and depressive symptoms. *S. intestinalis* is a species that has not been extensively studied, and its role in the gut microbiota is not fully understood. However, in an earlier study (Radjabzadeh et al., 2022), enrichment in the genus *Sellimonas* was associated with depressive symptoms, which is consistent with our results.

Our finding that women who displayed anxiety symptoms during the postpartum period, specifically at six and 12 months postpartum, had a higher abundance of *F. plautii* may be supported by the previous research, as the genus *Flavonifractor* has been shown to be linked to major depressive disorder (Zhong et al., 2022). The species *F. plautii* may contribute to depression via the degradation of quercetin which is a flavonoid with anti-inflammatory potential (Carlier et al., 2010). A recently published study showed that administration of quercetin alleviated depression-like behavior in mice and quercetin exerted an antioxidative effect in the hippocampus and prefrontal cortex (Ge et al., 2023). Thus, a higher abundance of *F. plautii* could increase the degradation of quercetin, potentially influencing the occurrence of depressive symptoms. Although, in the present study, higher abundances of *F. plautii* were associated with anxiety rather than depression. It is of note, that we observed several bacterial species which were associated with both depressive and anxiety symptoms, suggesting that the pathophysiology of perinatal depression and anxiety may share similar mechanisms. Thus, investigating the relation of the gut microbiota on comorbidity of perinatal depressive and anxiety symptoms would be of interest in the future studies.

The relative abundances of *S. parasanguinis* and *S. salivarius* were higher in women with depressive symptoms in early pregnancy. *S. parasanguinis* was also more abundant in early pregnancy in women with clinically significant anxiety symptoms at six and 12 months postpartum. *S. parasanguinis* and *S. salivarius* belong to the normal flora in the oral cavity; in previous studies, these were the species most frequently shared between the saliva and gut (Kageyama et al., 2023). *Streptococcus* in general has been found to be enriched in major depressive disorder (Lin et al., 2017). Additionally, a recent study in pregnant women with normal weight found that genus *Streptococcus* was positively associated with perinatal depressive and anxiety symptoms (Xu et al., 2025).

*H. mannosilyticum*, a species within the Ruminococcaceae family, showed increased relative abundance in women experiencing postpartum anxiety symptoms at three, six, and 12 months, as well as in those with postpartum depressive symptoms at three months. Relative abundances of *R. callidus*, another member of the Ruminococcaceae family, were found to be higher in women experiencing prenatal depressive symptoms, and in those with postpartum anxiety symptoms at three, six, and 12 months. A decrease in the abundance of *R.callidus* has been linked to depression in non-pregnant individuals (Maes et al., 2023), which is at odds with our findings. Unexpectedly, a higher abundance of *I. butyriciproducens* in late pregnancy was associated with postnatal depressive and anxiety symptoms, as *I. butyriciproducens* is a butyrate-producing bacteria, which has been considered to possess anti-depressant potential (Suda and Matsuda, 2022).

A previous study found that higher abundance of genus *Ca. Soleaferrea* in the third trimester was associated with a lower risk of prenatal depression in women with normal weight (Xie et al., 2024). Similarly, while another study in pregnant women found association between *Shigella* and postnatal depressive symptoms (Y. Zhou et al., 2020), we did not find an association between these bacteria and the symptom trajectories. Interestingly, Xie et al. (2024) pointed out that *Ca. Soleaferrea* has anti-inflammatory effects (Xie et al., 2024). Similarly, we noted that, some of the species enriched in women with postpartum depressive and anxiety symptoms, such as *H. hathewayi* and *B. clarus*, have been reported to link with increased inflammation. Thus, despite the deviating findings, it may well be that inflammation plays a role in the pathophysiology of depression and anxiety, but likely through different microbial pathways. While the specific bacterium previously linked to reduced depressive symptoms was not observed in our study, the presence of other inflammation-associated bacteria may indicate that inflammatory processes are a common underlying factor in depression and anxiety, regardless of the specific microbial composition. It is of note, that all the previous studies used 16S rRNA sequencing and analyzed data at the genus level, whereas the strength in our study is that we used shotgun metagenomic sequencing at species level, but at the same time making direct comparisons between the studies challenging. Another notable difference from prior studies is that the population in this study included only pregnant women with overweight and obesity, whereas the previous studies had only pregnant women with normal weight. It has been shown that the composition of the gut microbiota differs between pregnant women with overweight or obesity and normal weight (Dreisbach et al., 2019). Specifically, based on the systematic review by Dreisbach et al. (2019), e.g., three out of nine studies found that decrease in the abundance of species *C. coccoides* was associated with maternal obesity. Additionally, three out of five studies found lower α-diversity to be associated with maternal obesity. This is an emerging field of research and there are currently no previous studies investigating the differences in the gut-brain axis in pregnant women with normal weight and those who are living with overweight or obesity. Thus, including pregnant women with normal weight in the future studies would be beneficial in order to investigate the role of gut microbiota on the increased risk for depressive and anxiety symptoms in pregnant women with overweight and obesity.

Previous studies have pointed to a possible link between a lower α-diversity and symptoms of depression and anxiety in non-pregnant individuals (Y. Huang et al., 2018; Liu et al., 2016). However, some investigators have detected no difference in α-diversity between individuals with depression and healthy controls (Chahwan et al., 2019; Ritchie et al., 2023). We did not find any differences in the Shannon index between groups, in accordance with previous research in pregnant women (Xie et al., 2024). Thus, it is evident that more research is needed to clarify the contradictory results regarding changes in gut microbiota diversity.

The gut microbiota has been proposed to influence the brain through many direct and indirect pathways (Ullah et al., 2023). In terms of the chemical pathway, the gut microbiota can communicate via bacterial metabolites such as neurotransmitters and short-chain fatty acids. The gut microbiota can generate precursors of neurotransmitters or facilitate the synthesis of neurotransmitters through dietary metabolism (Chen et al., 2021). The neural pathway involves the most direct bidirectional route via the vagus nerve (Ullah et al., 2023). Our findings provide evidence supporting the theory of chemical signaling and inflammation. We demonstrated an association between elevated levels of bacteria linked to TMAO production and the subjects’ symptoms. TMAO has the capability to cross the blood-brain barrier (BBB), consequently impacting detrimentally on neurological and neuropsychiatric conditions (Mudimela et al., 2022). Additionally, we observed an association with the immune signaling pathway through the bacterial species associated with a pro-inflammatory environment in both depression and anxiety. However, depression and anxiety are complex disorders mediated by many factors, such as genetic susceptibilities (Norkeviciene et al., 2022). Therefore, more research is required not only on the gut microbiota but also on other contributing factors to gain a comprehensive understanding of these conditions, especially during pregnancy.

This study has several strengths with the most significant one being the utilization of metagenomic sequencing of high quality. In contrast to 16S rRNA sequencing, this method provides a more precise taxonomic resolution, enhancing the accuracy of our findings. Additionally, we have a well-characterized study population for whom we possess a wealth of background information, which allowed us to control the analyses for possible confounding factors. We found that frequency of smoking before pregnancy was highest in the High and decreasing depressive symptoms group and lowest in the Low and stable symptoms group. As previous research has revealed that the smoking may increase the risk of postpartum depression (Yook et al., 2022) as well as affect the gut microbiota composition (Antinozzi et al., 2022), we took a robust approach and adjusted the analyses for prepregnancy smoking. We also controlled the analysis with the original trial intervention group, as the consumption of fish oil and probiotics modified the gut microbiota composition of the participants to some extent as reported previously (Mokkala et al., 2021). Furthermore, it has been reported that symptoms of depression and anxiety exhibit variability and fluctuations throughout both the pre- and postnatal periods (Korja et al., 2018; Vänskä et al., 2011). We addressed this issue by utilizing symptom trajectories in addition to assessing symptoms at individual time points during the perinatal period. As the pregnant women with overweight and obesity are at increased risk for perinatal depressive and anxiety symptoms and overweight and obesity are common in Finland (THL, 2022), we chose to use the study population only with overweight or obesity and did not include participants with normal weight. When considering also other factors, for example age, our study population represents a very typical group of pregnant women in Finland.

The limitation of the study was that the group sizes of individuals suffering from depressive and anxiety symptoms was comparatively small, potentially limiting the statistical power. If the sample size had been larger, it might have been possible to observe even more bacterial species linked to depressive and anxiety symptom trajectories. In the cross-sectional analyses, we observed more species that differed statistically significantly between the study groups, likely because this approach focused on assessing the clinically significant depressive and anxiety symptoms at specific time points, while the trajectory analyses summarized course of symptoms over the perinatal period. Additionally, despite its numerous advantages, a longitudinal study design often suffers from dropouts, as also occurred in this study. The prepregnancy BMI was calculated based on the self-reported weight, which can be considered a limitation. However, we have confirmed the accuracy of self-reported weight previously within this study population, and have reported that there was a high correlation between self-reported weight and weight measured at fist study visit in early pregnancy (Saros et al., 2023).

It has been shown that maternal anxiety and depression, may affect not only maternal health but also the child’s developmental outcomes (Zhang et al., 2023b). One potential pathway could be through the maternal GBA. In this study, we investigated the initial phase of this pathway and demonstrated that elevated levels of certain pathogenic bacteria may contribute to depressive and anxiety symptoms not only during pregnancy but also in the postpartum period. In the future, it would be important to investigate the maternal gut microbiota in relation to the children’s developmental outcomes.

## CRediT authorship contribution statement

**Janina Hieta:** Conceptualization, Formal analysis, Writing – original draft. **Chouaib Benchraka:** Data curation, Formal analysis, Writing – review & editing. **Katariina Pärnänen:** Data curation, Writing – review & editing. **Noora Houttu:** Conceptualization, Data curation, Investigation, Supervision, Writing – review & editing. **Kati Mokkala:** Writing – review & editing. **Mrunalini Lotankar:** Writing – review & editing. **Eeva-Leena Kataja:** Writing – review & editing. **Leo Lahti:** Supervision, Writing – review & editing. **Kirsi Laitinen:** Conceptualization, Funding acquisition, Project administration, Supervision, Writing – review & editing.

## Data availability statement

The data sets are not available due to the fact that they contain information that could compromise the privacy and consent of the participants. The source code for the analyses is available online (https://doi.org/10.5281/zenodo.11471401).

## Role of the funding source

This work was supported by the State Research Funding for university-level health research in the Turku University Hospital Expert Responsibility Area, Academy of Finland (#258606), the Diabetes Research Foundation, the Juho Vainio Foundation, Signe and Ane Gyllenberg Foundation and Päivikki and Sakari Sohlberg Foundation.

## Declaration of competing interest

The authors declare that they have no known competing financial interests or personal relationships that could have appeared to influence the work reported in this paper.
