# Supplementary material for: Perinatal depressive and anxiety symptoms are associated with gut microbiota in pregnant women with overweight and obesity
Source: Brain Behav Immun Health. 2025 Jun 19;47:101042. doi: 10.1016/j.bbih.2025.101042 (PMC12246855; doi:10.1016/j.bbih.2025.101042)
Supplement: Multimedia component 2 [file mmc2.docx]

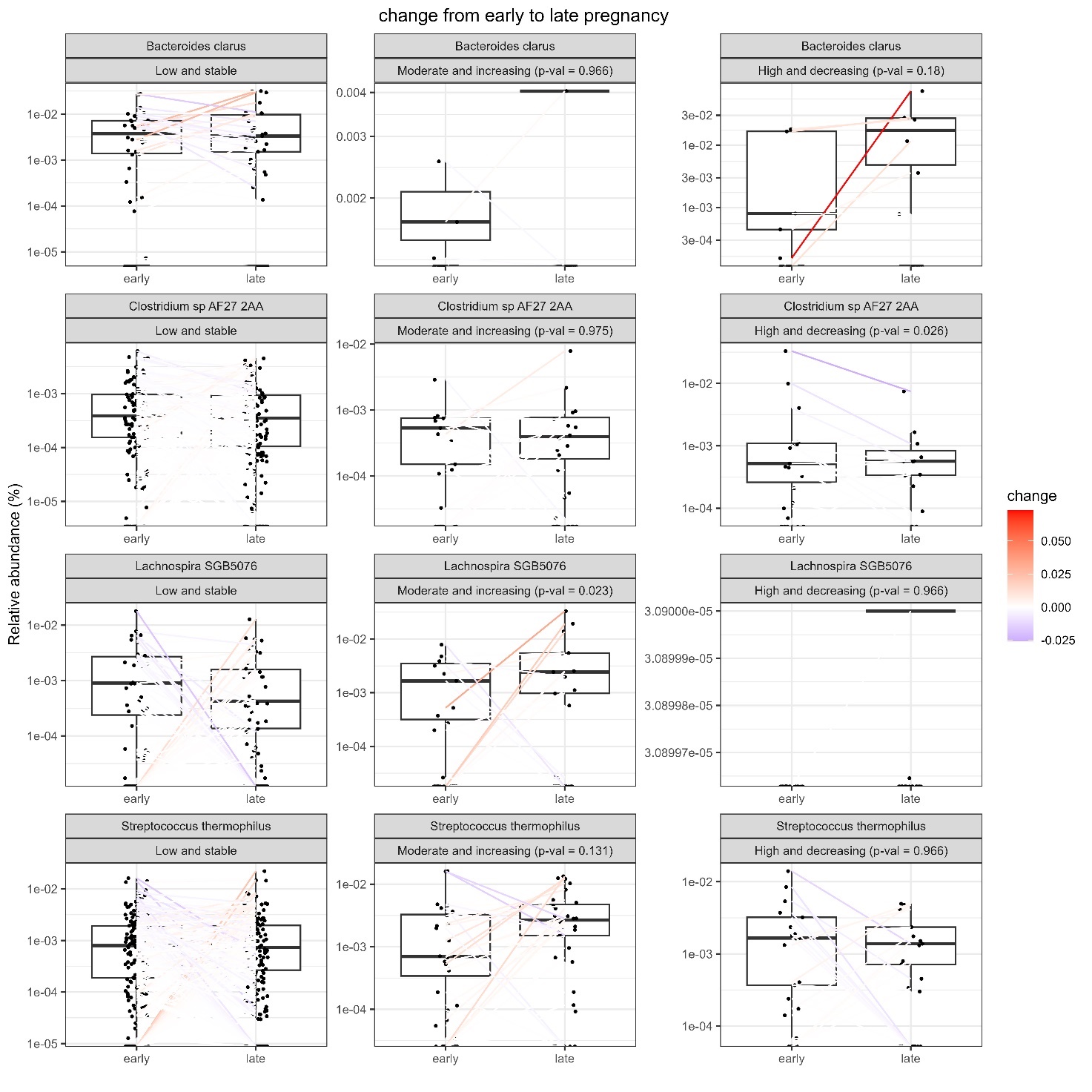


Supplementary figure S1. A comparison of the change in relative abundances of the species with significant (FDR<0.05) or borderline significant (FDR<0.25) differences from early to late pregnancy between the women who had depressive symptoms in the perinatal period and those women who did not. The significance was estimated with MaAsLin2 with a prevalence of 10% and detection limit on relative abundance data of 1e-4. The following covariates were included in the model: intervention, IDQ (index of diet quality), prepregnancy BMI (Body Mass Index) and smoking status before pregnancy.


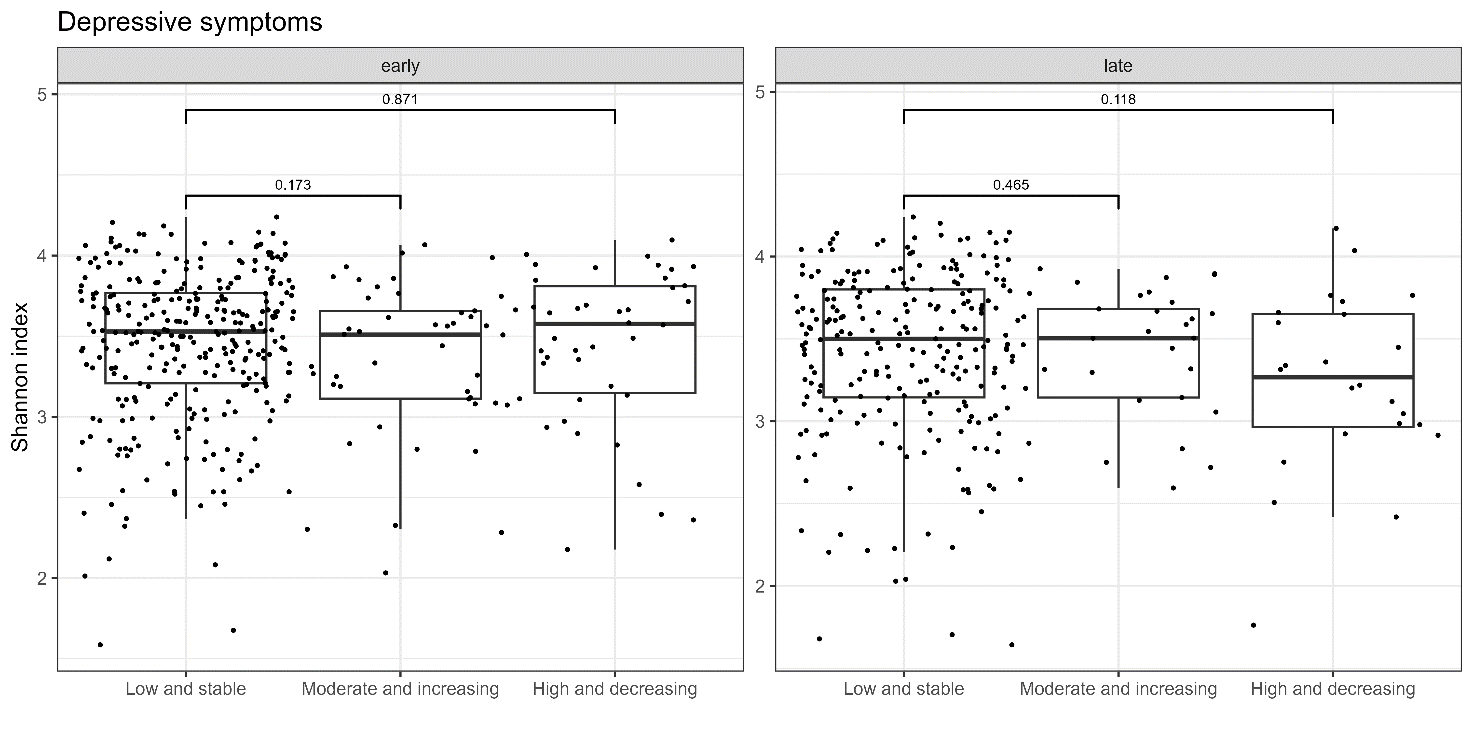
Supplementary figure S2. A comparison of the α-Diversity (Shannon index) in early and late pregnancy in women with low and stable, moderate and increasing or high and decreasing depressive symptoms. No significant differences were observed with the linear model adjusted with the covariates; prepregnancy BMI (Body Mass Index), intervention group, Index of Diet Quality (IDQ) and smoking status before pregnancy.


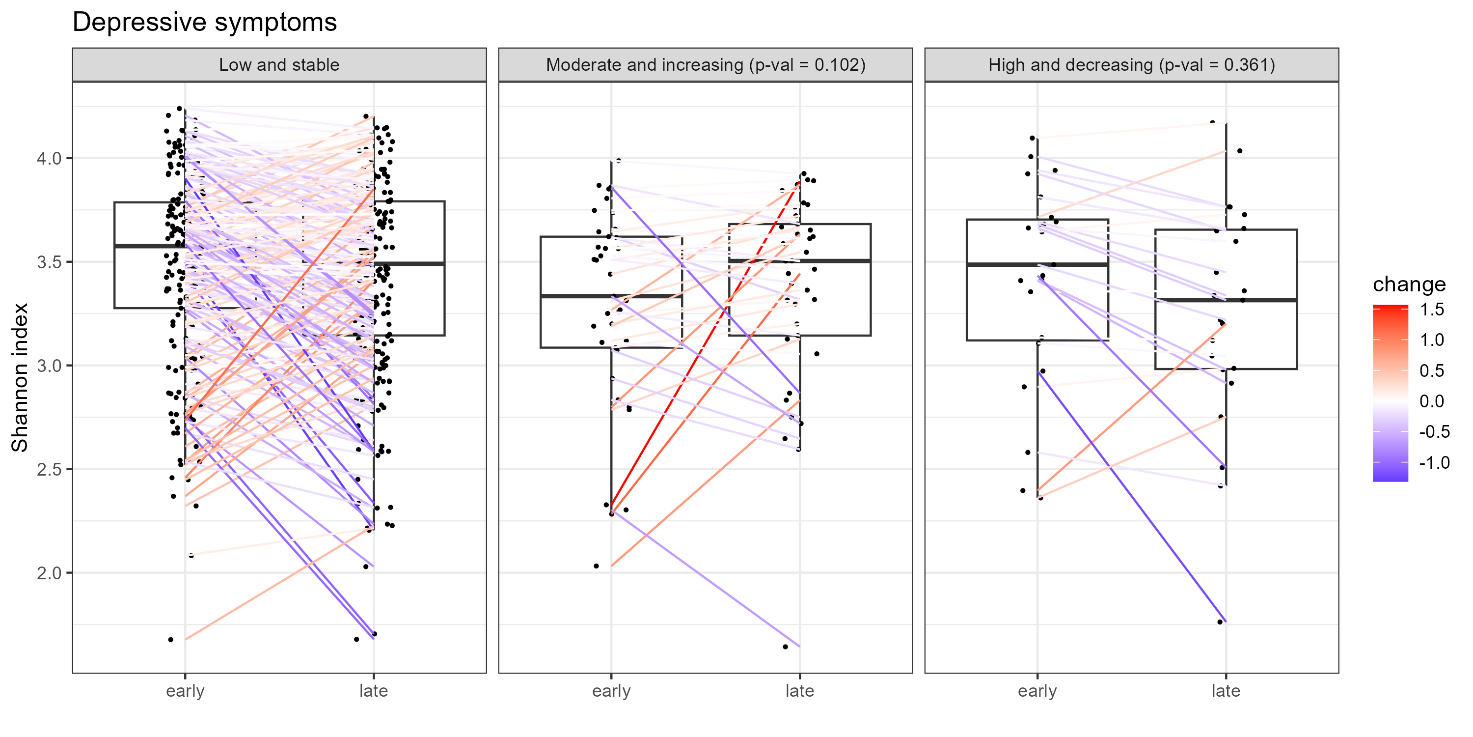
Supplementary figure S3. A comparison of the change in α-Diversity (Shannon index) from early to late pregnancy in women with low and stable, moderate and increasing or high and decreasing depressive symptoms. No significant differences were observed with the linear model adjusted with the covariates; prepregnancy BMI (Body Mass Index), intervention group, Index of Diet Quality (IDQ) and smoking status before pregnancy.


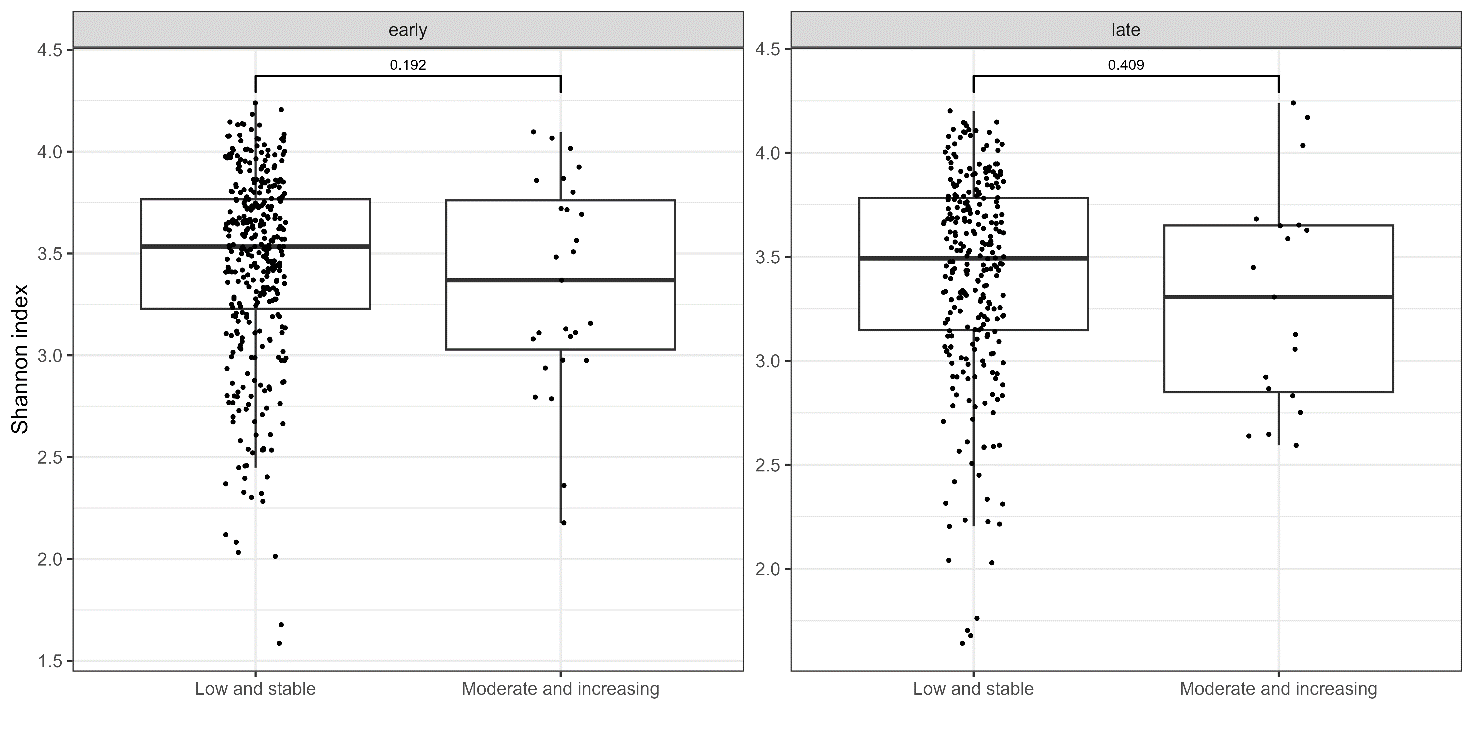
Supplementary figure S4. A comparison of the α-Diversity (Shannon index) in early and late pregnancy in women with low and stable or moderate and increasing anxiety symptoms. No significant differences were observed with the linear model adjusted with the covariates; prepregnancy BMI (Body Mass Index), intervention group, Index of Diet Quality (IDQ) and smoking status before pregnancy.


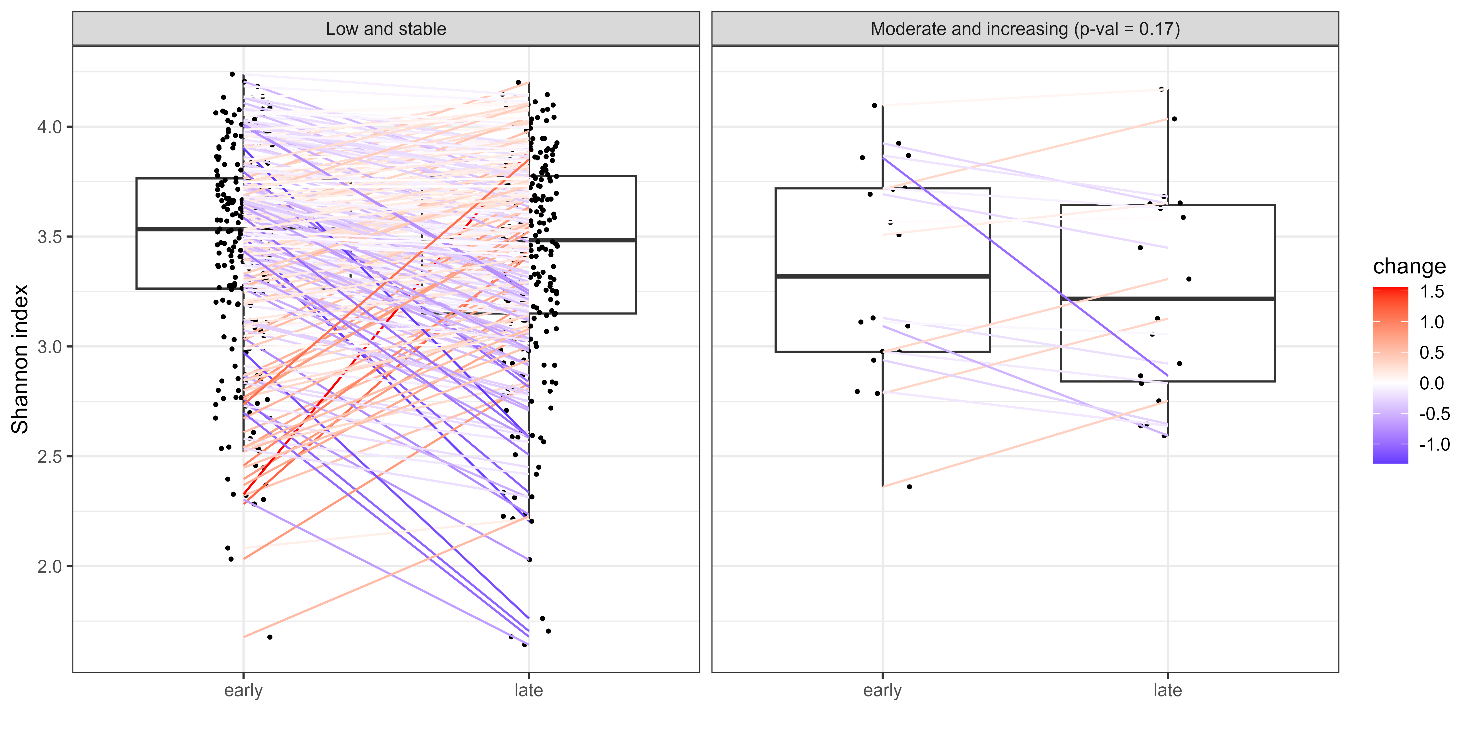

Supplementary figure S5. A comparison of the change in α-Diversity (Shannon index) from early to late pregnancy in women with low and stable or moderate and increasing anxiety symptoms. No significant differences were observed with the linear model adjusted with the covariates; prepregnancy BMI (Body Mass Index), intervention group, Index of Diet Quality (IDQ) and smoking status before pregnancy.
